# Experimental Researches on the Nutritive Power of Green and Dry Fodder

**Published:** 1847-02

**Authors:** 


*Experimental Researches on the Nutritive Power of Green and Dry Fodder.* By M. Boussingault, (Ann. de Chim. et de Phys., July, 1846, and Comptes Rendus, Apr. 1846.)—It is generally thought that there is more nourishment in green fodder than in the dry hay derived from an equal weight of the fresh grass. The experiments of Boussingault show that this is not true. A heifer was weighed, and fed for ten days on green fodder, each day a quantity equal in weight to that consumed was put aside to dry. The animal was again weighed and fed for ten days on the dry fodder, then weighed again. The experiment was tried three times, and each time the animal weighed a little more after feeding on the dry fodder than after the green. The difference was not enough to prove that the dry food was the *more* nutritious, although the experiment proved beyond a doubt that it was not inferior in effect to the other.—*Amer. Jour. Sci.*

